# Stable Cu_2_O/TiO_2_ nanorod photoelectrodes for enhanced photoelectrochemical water splitting in ethanol-assisted systems

**DOI:** 10.1016/j.isci.2026.117997

**Published:** 2026-10-28

**Authors:** Srijitra Khanpakdee, Teera Butburee, Supphasin Thaweesak, Jung-Ho Yun, Miaoqiang Lyu, Piangjai Peerakiatkhajohn

**Affiliations:** 1 Faculty of Environment and Resource Studies, Mahidol University, Nakhon Pathom 73170, Thailand; 2 National Nanotechnology Center, National Science and Technology Development Agency, 111 Thailand Science Park, Pathum Thani 12120, Thailand; 3 Department of Chemical Engineering, Faculty of Engineering, Burapha University, Chon Buri 20131, Thailand; 4 Department of Environmental Science and Engineering, College of Engineering, Kyung Hee University, 1732 Deogyeong-daero, Giheung-gu, Yongin-si, Gyeonggi-do 17104, Republic of Korea; 5 Nanomaterials Centre, School of Chemical Engineering and Australian Institute for Bioengineering and Nanotechnology (AIBN), The University of Queensland, St Lucia, QLD 4123, Australia

**Keywords:** photoelectrochemical, hydrogen, photoelectrode, ethanol, heterojunction

## Abstract

The heterojunction of the Cu_2_O/TiO_2_ nanorod photoelectrode was synthesized via a facile hydrothermal method and electrodeposition method. The influence of Cu_2_O deposition on the structural, optical, and photoelectrochemical properties of the photoelectrodes was systematically investigated through comprehensive physicochemical characterization. Under AM 1.5 simulated solar illumination, the optimized Cu_2_O/TiO_2_ nanorod photoelectrodes achieved a photocurrent density of 1.30 mA cm^−2^ at 1.23 V vs. RHE in an ethanol-containing electrolyte. The enhanced photoelectrochemical performance was attributed to improved light utilization and more efficient charge separation with suppressed electron-hole recombination resulting from the formation of the Cu_2_O/TiO_2_ heterojunction. Simultaneously, ethanol also served as a sacrificial electron donor in the proposed system. Notably, the Cu_2_O/TiO_2_ nanorod photoelectrodes can be promising photoelectrodes for solar-driven hydrogen production with a simple, economical, and environmentally friendly approach.

## Highlights

• Cu_2_O was electrodeposited onto hydrothermally grown TiO_2_ nanorods
• Cu_2_O loading tuned the structure, optical properties, and PEC performance
• Cu_2_O/TiO_2_ reached 1.30 mA cm^−2^ at 1.23 V vs. RHE with stable performance
• Ethanol acted as a sacrificial donor to enhance solar H_2_ production

## Graphical abstract

**Figure:**
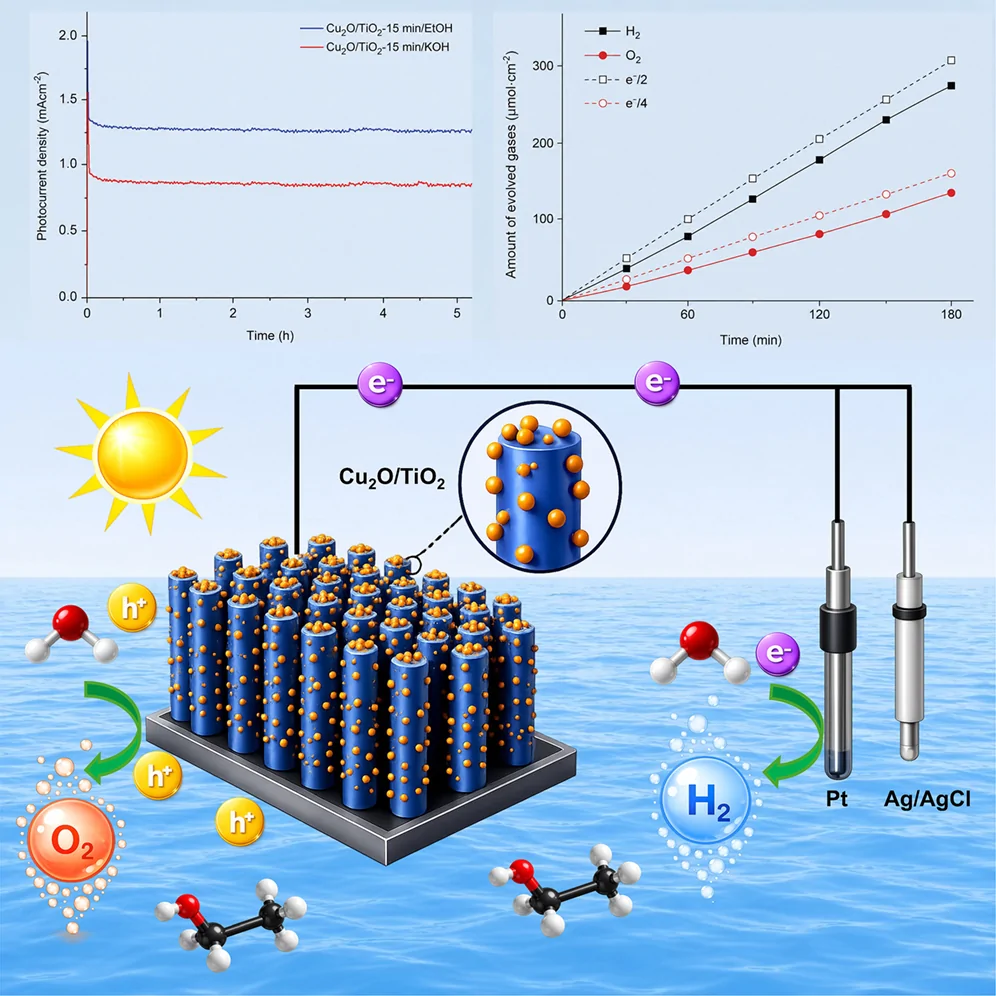

## Teaser

catalysis; electrochemistry; environmental chemical engineering

## Introduction

The growing global energy demand driven by population expansion and industrial development continues to rely heavily on fossil fuels, leading to severe environmental impacts, particularly greenhouse gas emissions and climate change.^1^^,^^2^ Hydrogen (H_2_) is widely regarded as a key clean energy carrier due to its carbon-free end use and compatibility with existing energy infrastructure. However, current H_2_ production is dominated by “gray” and “blue” routes based on fossil fuel reforming, which generate unavoidable carbon dioxide (CO_2_) emissions.^3^^,^^4^ Meanwhile, green hydrogen remains limited by high costs, intermittent renewable energy supply, and scalability challenges. Photoelectrochemical (PEC) water splitting is a promising and sustainable approach to hydrogen production, using solar energy to drive the splitting of water into hydrogen and oxygen.^5^^,^^6^^,^^7^^,^^8^ However, the practical application of PEC systems requires efficient, durable, and cost-effective photoelectrodes that can efficiently convert sunlight into chemical energy while maintaining long-term stability.^9^^,^^10^^,^^11^ A key requirement for PEC photoelectrodes is their ability to absorb light across a broad spectrum, particularly in the visible region, as well as to exhibit excellent charge carrier mobility and minimal recombination. Additionally, these photoelectrodes must be chemically stable under prolonged illumination and exposure to the corrosive conditions present during water splitting reactions.^12^^,^^13^^,^^14^ The H_2_ production in PEC system has emerged as a promising solar-driven alternative,^15^^,^^16^ with recent attention shifting toward bifunctional PEC systems that couple H_2_ evolution with organic oxidation reactions.^17^^,^^18^ Compared with conventional H_2_ evolution, organic oxidation offers significantly lower thermodynamic barriers, reduced overpotentials,^19^ and the generation of value-added chemicals,^20^ while simultaneously enabling wastewater remediation.^21^ These advantages position bifunctional PEC systems as an attractive strategy for efficient and sustainable solar fuel production. Davies et al.^22^ recently demonstrated an efficient bifunctional PEC system using a mesoporous WO_3_/BiVO_4_ photoanode, which could simultaneously achieve complete degradation of anionic and cationic surfactants while generating of green H_2_. These findings highlight the versatility of bifunctional solar-driven platforms as a superior alternative for integrated energy production and environmental remediation. Beyond system configuration, the efficiency of PEC platforms is fundamentally governed by the properties of the semiconductor photoelectrodes.^23^^,^^24^ Earth-abundant and cost-effective metal oxide semiconductors, such as TiO_2_,^25^^,^^26^^,^^27^ ZnO,^28^^,^^29^^,^^30^ WO_3_,^31^^,^^32^^,^^33^ Fe_2_O_3_,^16^^,^^34^^,^^35^ Cu_2_O,^36^^,^^37^^,^^38^ and BiVO_4_,^31^^,^^39^^,^^40^ have been widely studied as photocatalysts in PEC systems.^13^^,^^41^^,^^42^ Despite these options, titanium dioxide (TiO_2_) remains one of the most widely used materials due to its exceptional chemical stability, low cost, and superior environmental compatibility.^43^^,^^44^ However, its wide bandgap restricts light absorption primarily to the ultraviolet (UV) region and often results in severe charge carrier recombination.^45^^,^^46^^,^^47^ To address these limitations, cuprous oxide (Cu_2_O), a p-type semiconductor with strong visible-light absorption,^48^ has been employed as an effective complement; however, it suffers from photocorrosion and rapid charge recombination under illumination, leading to a significant reduction in its efficiency and stability.^37^^,^^49^ Nanostructuring these heterojunctions into nanorods (NRs) further enhances their surface area, providing more active sites for light absorption and catalytic reactions, which is crucial for improving PEC performance. Constructing Cu_2_O/TiO_2_ p-n heterojunctions combines the stability of TiO_2_ with the visible-light activity of Cu_2_O, creating an internal electric field at the heterointerface that promotes charge separation.^37^^,^^50^ When integrated with one-dimensional (1D) TiO_2_ nanostructures, these heterojunctions further enhance light harvesting and charge transport,^51^ making them particularly suitable for high-performance bifunctional PEC applications. Despite extensive studies on Cu_2_O/TiO_2_ heterostructures, the influence of Cu_2_O deposition amount and structural configuration on interfacial band alignment, junction quality, and charge recombination behavior has not yet been fully elucidated. In addition to structural modifications, the use of a sacrificial agent or organic substances such as dye pollutant^52^^,^^53^^,^^54^ and organic substances^17^^,^^55^^,^^56^^,^^57^ has recently been introduced in bifunction PEC systems. Ethanol (EtOH) is regarded as an attractive, non-toxic compound as an electrolyte because of its well-established supply chain, carbon-neutral production from agricultural biomass, high theoretical energy density, and straightforward storage and infrastructure requirements.^58^^,^^59^^,^^60^ Moreover, most existing research has focused on PEC water splitting, while systematic investigations into bifunctional PEC systems operating in ethanol-containing electrolytes are still limited. In particular, the structure-property-performance relationships governing simultaneous hydrogen evolution and ethanol oxidation have not been clearly established, hindering rational design and optimization of Cu_2_O/TiO_2_-based photoelectrodes for bifunctional PEC applications. This study aims to develop Cu_2_O/TiO_2_ heterostructured photoelectrodes via a facile hydrothermal method and to systematically correlate synthesis conditions with physicochemical properties and PEC performance. The work demonstrates the coupled hydrogen generation and ethanol degradation within a single PEC system and provides mechanistic insight into interfacial charge transfer and band alignment. The novelty lies in elucidating how Cu_2_O deposition governs heterojunction functionality under bifunctional PEC operation, offering a low-cost and sustainable strategy for integrated solar-driven hydrogen production and organic oxidation.

## Results

### Characterization of TiO_2_ and Cu_2_O/TiO_2_ photoelectrodes

Figures 1A–1H shows the surface morphology and cross-sectional FE-SEM images of TiO_2_ and Cu_2_O/TiO_2_ photoelectrodes synthesized via hydrothermal reaction with different electrodeposition durations. The TiO_2_ photoelectrode exhibits a densely and uniformly vertical growth pattern with a 1D NRs, having larger average widths of ∼235 nm and lengths ∼6.3 *μ*m. It can be confirmed that the facile hydrothermal method in this study provided a compact arrangement and well-formed nanostructure with significant vertical alignment, suitable for enhancing charge transport and photocatalytic performance. In addition, the electrodeposition of Cu_2_O layer on TiO_2_ photoelectrode with different deposition times, the Cu_2_O/TiO_2_ photoelectrodes reveal a densely packed NR structure with a continuous thin layer Cu_2_O coverage can be observed over the TiO_2_ surface, with the TiO_2_ NRs remaining distinct. This configuration is likely to offer good photocatalytic activity and charge separation efficiency. As the deposition time increases, the Cu_2_O coverage becomes denser and more pronounced. This may enhance the p-n junction formation and potentially improve charge carrier dynamics and charge separation behavior.^36^^,^^38^

**Figure 1:**
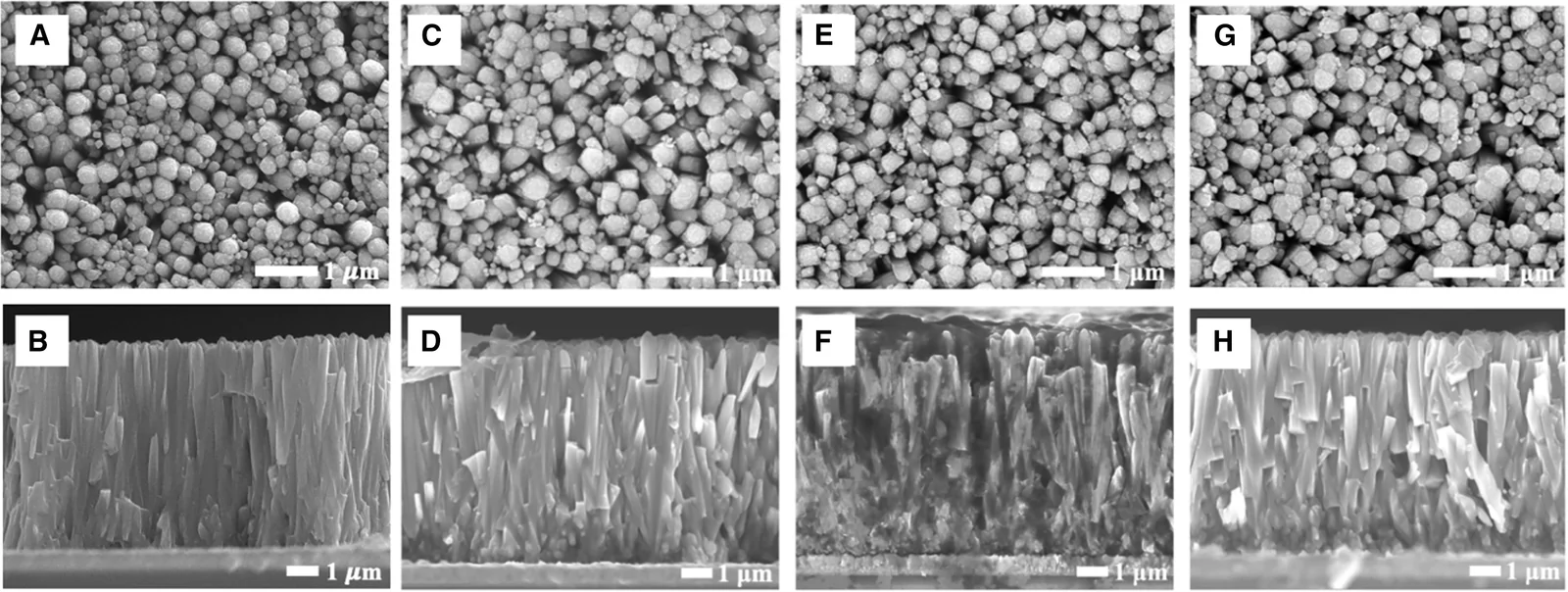
FE-SEM top-view and cross-sectional images depicting the morphology of (A and B) TiO_2_, (C and D) Cu_2_O/TiO_2_ -5 min, (E and F) Cu_2_O/TiO_2_ -10 min and (G and H) Cu_2_O/TiO_2_ -15 min photoelectrodes. Scale bars, 1 μm in all figures

The EDS spectra and elemental mapping presented in Figure 2A provide insights into the elemental distribution within the Cu_2_O/TiO_2_ photoelectrode. The elemental maps for Ti and O exhibit a uniform distribution, indicating the predominant presence of TiO_2_ across the sample. In contrast, the elemental map for Cu shows that Cu is thin later throughout the photoelectrode, suggesting that the Cu_2_O deposits are confined to localized regions. The EDS spectra confirm the presence of Cu, revealing an average weight percentage of approximately 0.3% across all samples (Cu_2_O/TiO_2_-5 min, Cu_2_O/TiO_2_-10 min, and Cu_2_O/TiO_2_-15 min). Figure 2B presents the XRF analysis of the Cu_2_O/TiO_2_ NR. The analysis indicates that Ti is the dominant element, reflecting the consistent formation of the TiO_2_ layer. Additionally, the Cu content (wt%) shows a gradual increase with longer deposition times. This trend suggests effective deposition of Cu_2_O onto the TiO_2_ layer with increased deposition time. Elemental mapping further confirms the uniform distribution of Ti and Sn, while Cu appears sparsely dispersed, consistent with its minor concentration in the films.

**Figure 2:**
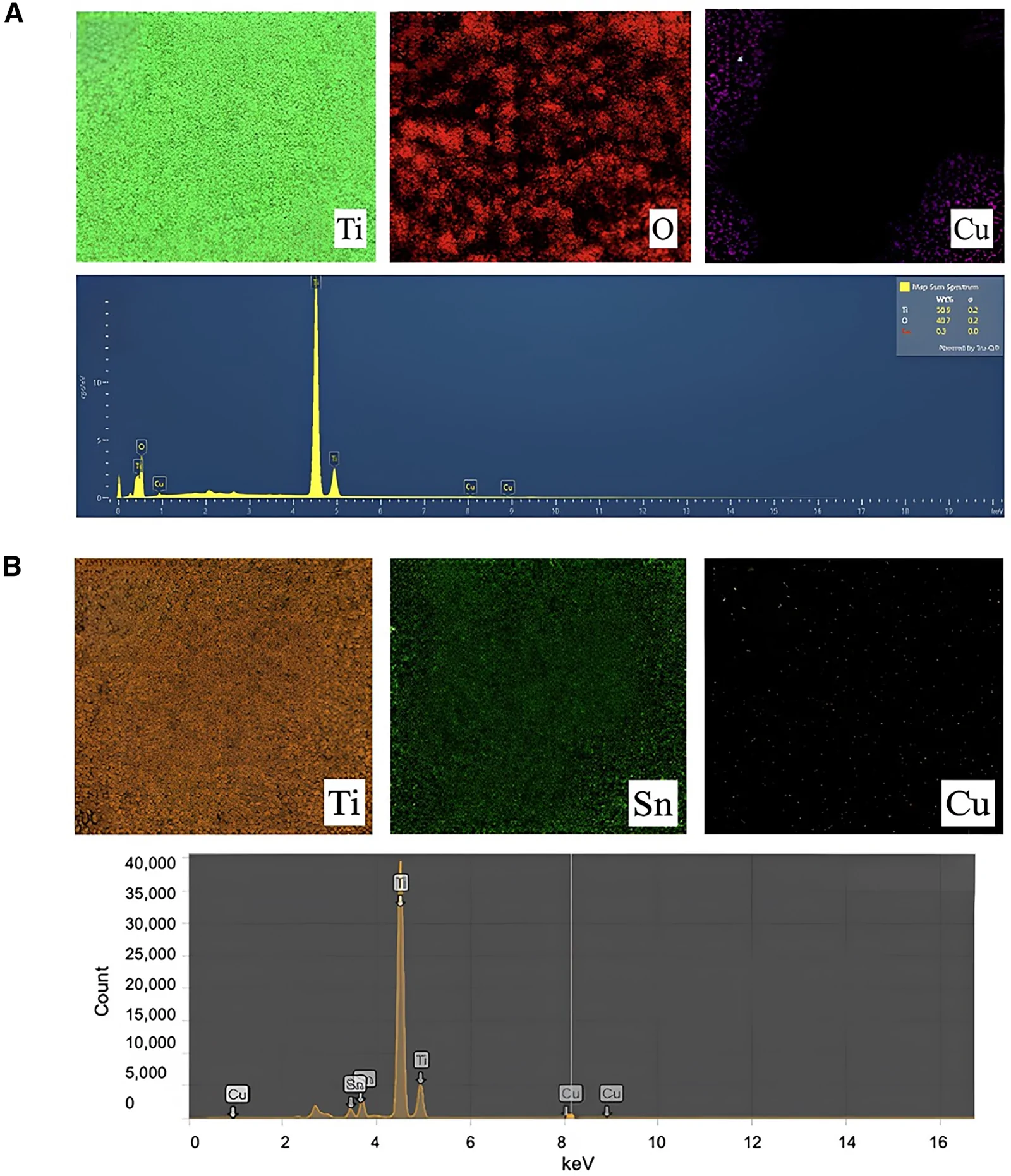
(A) EDS spectra and elemental mapping (B) XRF analysis of the Cu_2_O/TiO_2_-15 min photoelectrode

The TEM and HRTEM images in Figures 3A–3F of the Cu_2_O/TiO_2_ heterostructures reveal significant structural details, highlighting the single-crystalline nature of the TiO_2_ NRs. The lattice fringes observed in the HRTEM images confirm the high crystallinity of the TiO_2_ phase, showcasing well-defined spacing consistent with its crystal planes. Notably, a d-spacing of 0.17 nm is identified, corresponding to the (211) plane of rutile TiO_2_, further emphasizing its crystalline structure. Additionally, the Cu_2_O particles are distinctly visible, dispersed on the surface of the TiO_2_ NRs. The presence of these Cu_2_O particles is corroborated by their distinct lattice fringes, confirming the successful formation of the Cu_2_O/TiO_2_ heterostructure. The intimate contact between the Cu_2_O particles and TiO_2_ NRs promotes efficient charge transfer.

**Figure 3:**
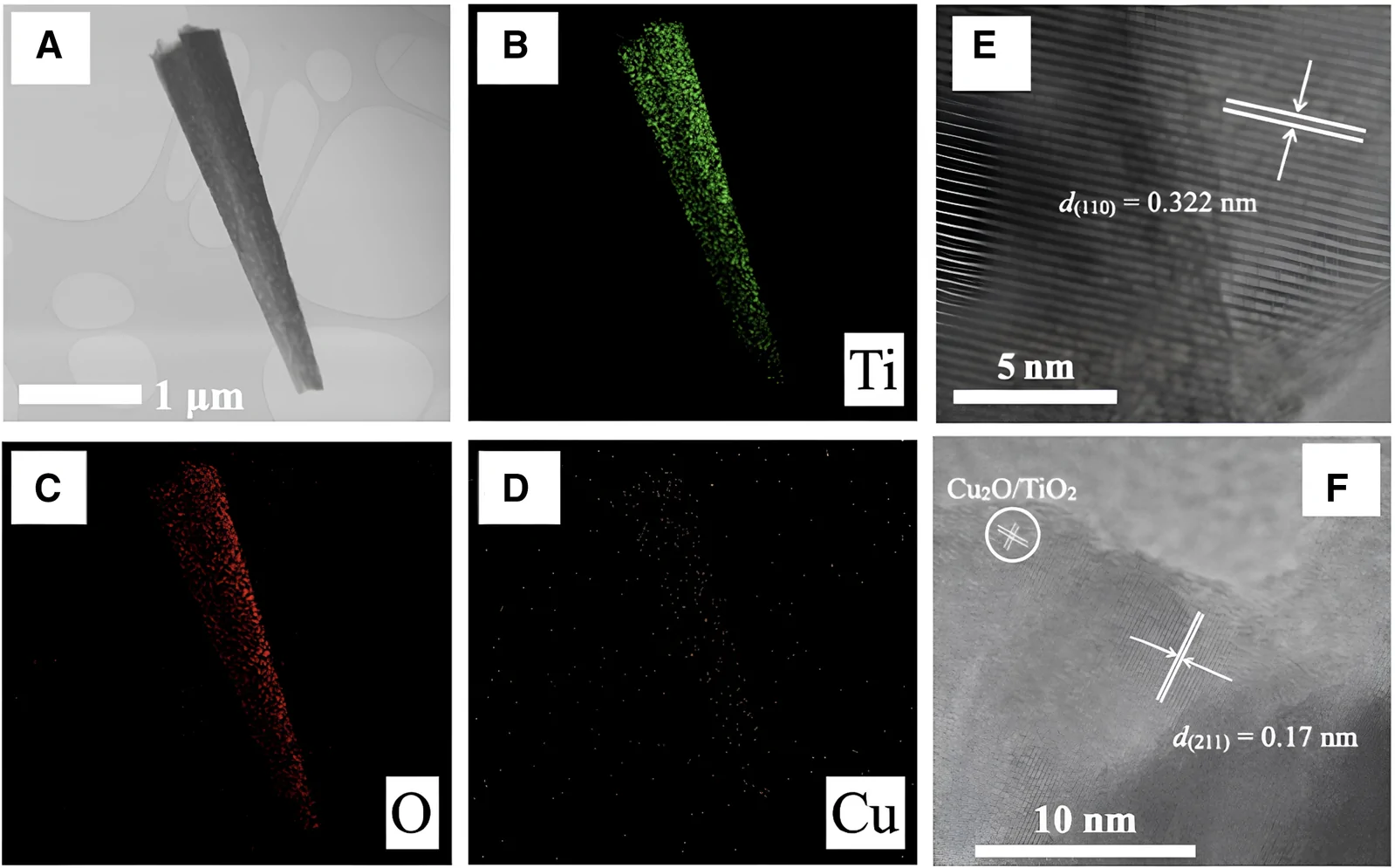
TEM image of the Cu_2_O/TiO_2_ NRs with corresponding elemental maps for (B) Ti, (C) O, and (D) Cu, and HRTEM images of (E) TiO_2_ NRs and (F) Cu_2_O/TiO_2_ NR photoelectrodes

The XRD patterns in Figure 4A illustrate the crystalline structures of TiO_2_ and Cu_2_O/TiO_2_ NRs deposited on FTO substrates. The diffraction peaks located at 2θ values of 36.1°, 41.3°, 54.4°, 56.1°, 62.9°, and 70° correspond to the (101), (111), (211), (221), (002), and (112) planes of rutile TiO_2_ (JCPDS# 01–087–0710), confirming the formation of a well-crystallized rutile phase.^61^ A weak diffraction peak observed near 29.5° is attributed to the (110) plane of Cu_2_O (JCPDS# 00–005–0667), suggesting the successful deposition of Cu_2_O onto the TiO_2_ NRs.^36^^,^^38^ The relatively low intensity of the Cu_2_O peak compared to TiO_2_ is likely due to the surface-localized distribution of Cu_2_O, while the strong rutile TiO_2_ diffraction dominates the overall XRD pattern. Minor variations in Cu_2_O peak intensity among different deposition times may reflect differences in Cu_2_O coverage or local distribution on the TiO_2_ surface. Among the Cu_2_O/TiO_2_ samples, slight variations in the Cu_2_O (110) diffraction peak intensity were observed with different deposition times. The Cu_2_O/TiO_2_-10 min sample exhibited the most noticeable Cu_2_O-related peak, suggesting a relatively more uniform or favorable surface distribution of Cu_2_O on the TiO_2_ NRs. In contrast, the weaker Cu_2_O signals observed for the 5 min and 15 min samples may be associated with insufficient surface coverage at shorter deposition times and less uniform local deposition at longer deposition times. Nevertheless, the overall weak Cu_2_O diffraction intensity in all samples suggests that the deposited Cu_2_O is likely present as a finely dispersed surface phase on the TiO_2_ NRs rather than as a bulk crystalline phase.

**Figure 4:**
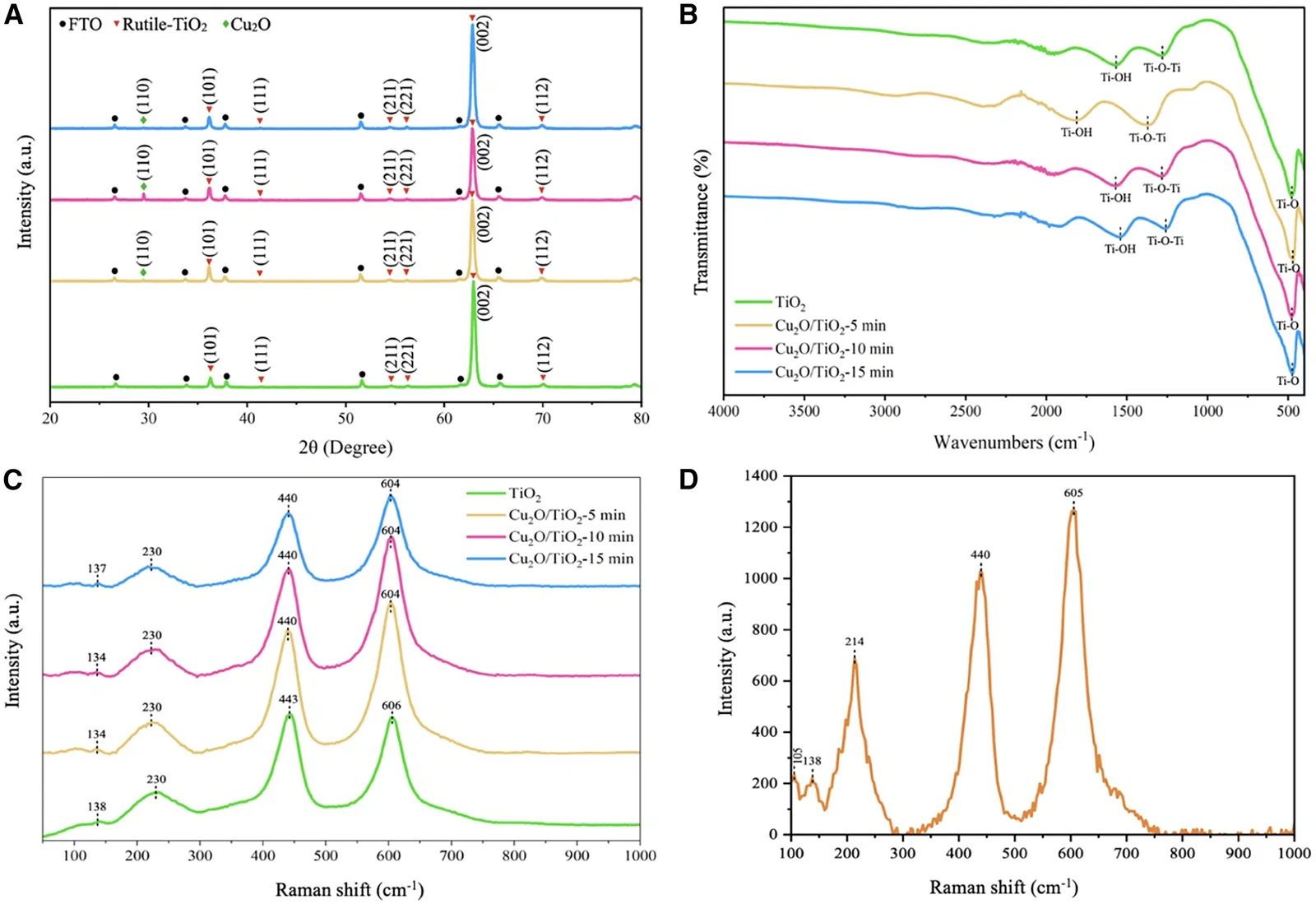
(A) XRD patterns, (B) FTIR spectra, and (C) Raman spectra of TiO_2_ NRs and Cu_2_O/TiO_2_ photoelectrodes with different of deposition time and (D) Raman spectra of Cu_2_O/TiO_2_ photoelectrodes focused on Cu_2_O-specific regions

The FTIR spectra presented in Figure 4B provide insight into the functional groups and bonding structures within the TiO_2_ and Cu_2_O/TiO_2_ samples. The FTIR spectra of TiO_2_ NRs, showing characteristic absorption bands at 1,570.45 cm^−1^ is associated with Ti-OH bond vibrations. Additional peaks at 1,284.81 cm^−1^ and 478.80 cm^−1^ correspond to Ti-O–Ti bond vibrations and Ti-O bending modes, respectively. All Cu_2_O/TiO_2_ samples exhibit characteristic peaks associated with Ti-OH, Ti-O-Ti, and Ti-O bonds. The peak around 1,500–1,800 cm^−1^ corresponds to the Ti-OH stretching vibrations. The Ti-O-Ti peaks, observed around 1,200–1,300 cm^−1^, indicate TiO_2_ network formation, essential for structural stability. Additionally, prominent Ti-O peaks appear around 400–500 cm^−1^, confirming the presence of the TiO_2_ phase in all samples.^37^ The FTIR data confirm the presence of the TiO_2_ phase but do not provide distinct evidence for Cu_2_O. The Raman analysis of TiO_2_ and Cu_2_O/TiO_2_ photoelectrodes, as shown in Figure 4C, provides significant insights into their vibrational properties and structural composition. For TiO_2_ photoelectrode it is obvious from the spectra that peaks appear at 138, 230, 442, and 606 cm^−1^, which peaks at 138, 442, and 606 cm^−1^, corresponding to the B_1g_, E_g_, and A_1g_ modes of the rutile phase, respectively. The TiO_2_ exhibit peaks at 138, 230, and 606 cm^−1^, with the 443 cm^−1^ peak. The B_1g_, and E_g_ modes originate from the symmetric bending vibration and the symmetric stretching vibration of O-Ti–O bonds, respectively. The A_1g_ modes appear from the antisymmetric bending vibration of Ti-O–Ti bonds. Additionally, the presence of multiple phonons scattering at 230 cm^−1^ suggests the nanometer scale of the TiO_2_ NRs.^38^ These results are consistent with the XRD analysis, which confirms the rutile phase.

The Raman spectra shown in Figure 4C are dominated by the characteristic vibrational modes of rutile TiO_2_, including the B_1g_, E_g_, and A_1g_ modes located near 138, 440, and 605 cm^−1^, respectively, together with the multiple-phonon scattering feature near 230 cm^−1 50, 58^. These results are consistent with the XRD analysis and confirm the rutile structure of TiO_2_ NRs. No distinct Cu_2_O-related Raman peaks are clearly visible in the full spectral range due to the dominant TiO_2_ signals and the relatively low Cu_2_O content. To further examine the presence of Cu_2_O, the Raman spectra were analyzed in Cu_2_O-specific regions, as shown in Figure 4D. Weak Raman features observed near 105 and 214 cm^−1^ are attributed to Cu_2_O-related vibrational modes.^36^ In particular, the peak near 214 cm^−1^ corresponds to the second-order Raman mode characteristic of Cu_2_O, supporting the presence of Cu_2_O within the heterostructure. These findings suggest that Cu_2_O exists as a thin or locally distributed surface layer on the TiO_2_ NRs, which explains why its signals appear weak in both XRD and the full-range Raman spectra.

To investigate the surface chemical composition and oxidation states of the Cu_2_O/TiO_2_ NR photoelectrode, XPS measurements were performed. The high-resolution spectra of Cu 2p, Ti 2p, and O 1s regions are shown in Figures 5A–5C. The Cu 2p spectrum exhibits two dominant peaks centered at approximately 932.8 and 952.6 eV, corresponding to Cu 2p_3/2_ and Cu 2p_1/2_, respectively. The absence of a strong satellite structure typical of CuO suggests that Cu^+^ is the predominant oxidation state on the electrode surface. The Ti 2p spectrum displays two well-defined peaks located at approximately 458.8 and 464.5 eV, which are assigned to Ti 2p_3/2_ and Ti 2p_1/2_, respectively. This result suggests that the electrodeposition process preserves the crystalline and electronic structure of TiO_2,_ thereby maintaining its role as an efficient electron transport layer in the heterojunction. The major peak at approximately 531.3 eV is attributed to lattice oxygen (O^2−^) associated with Ti-O and Cu-O bonds within the oxide framework (Biesinger et al., 2010). A second component centered around 532.4 eV is assigned to surface hydroxyl groups and chemisorbed oxygen species, whereas the weak peak near 530.0 eV can be associated with oxygen-deficient regions or oxygen-vacancy-related species. The presence of surface hydroxyl groups is advantageous for PEC water oxidation because hydroxylated surfaces enhance water adsorption and provide active sites for hole transfer reactions. In addition, oxygen vacancies can introduce shallow electronic states that enhance charge separation and suppress electron-hole recombination, thereby improving photocatalytic and PEC performance.^50^ These results verify the successful formation of the Cu_2_O/TiO_2_ heterojunction and the coexistence of surface species that may contribute to enhanced PEC activity.

**Figure 5:**
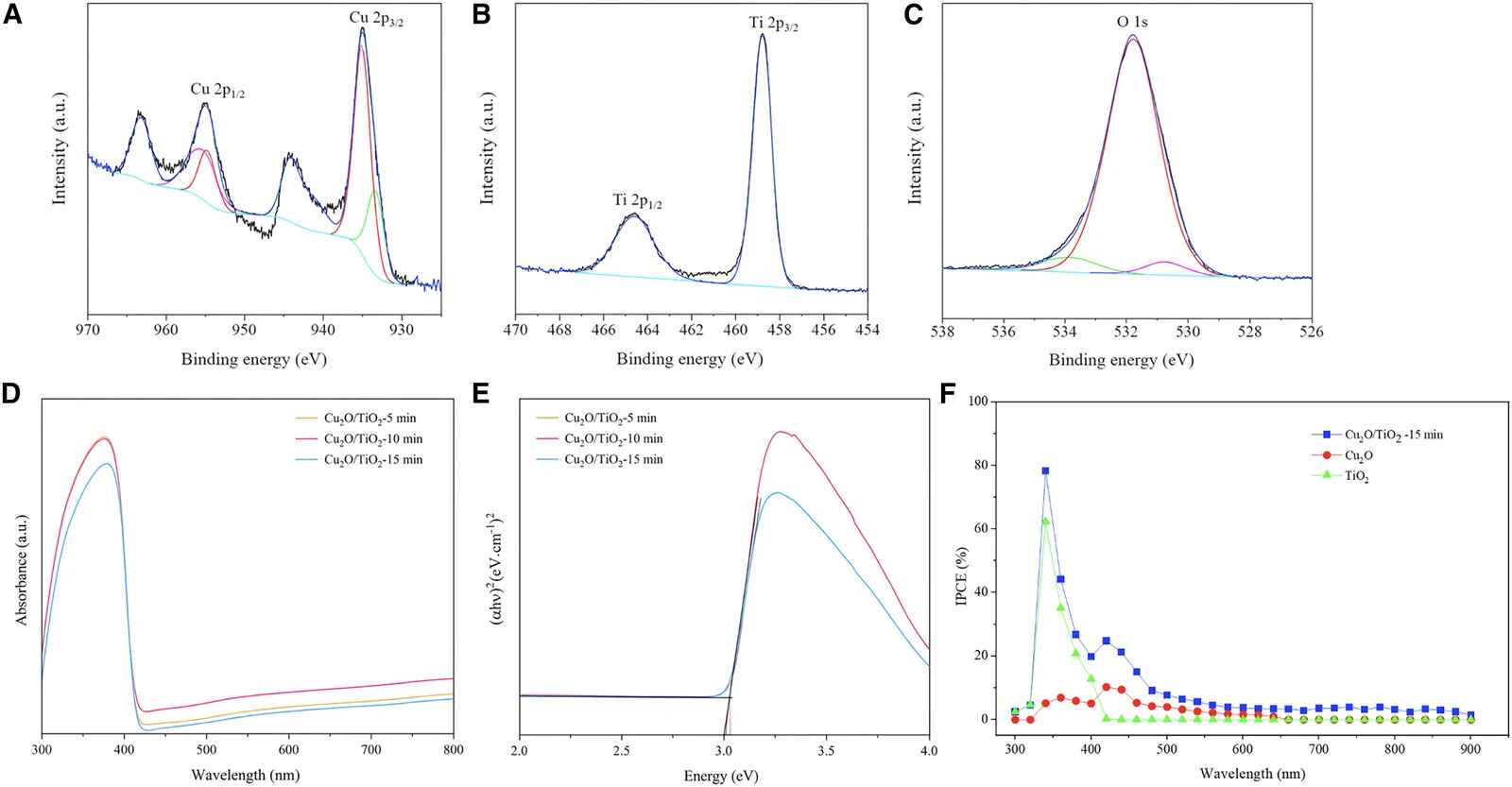
High-resolution XPS spectra of the Cu_2_O/TiO_2_ photoelectrode: (A) Cu 2p, (B) Ti 2p, and (C) O 1s regions, (D) UV-Vis absorbance spectra (E) Tauc plot of Cu_2_O/TiO_2_ photoelectrodes and (F) Incident photon-to-current conversion efficiency (IPCE) spectra of pristine TiO_2_, Cu_2_O, and Cu_2_O/TiO_2_-15 min photoelectrodes

Figures 5D and 5E displays the UV-Vis absorbance spectra and Tauc plot for 1 photoelectrode. In the absorbance spectra, all samples show a prominent absorption edge near 410 nm, which is characteristic of the rutile TiO_2_ phase and corresponds to its band gap in the UV region.^37^^,^^38^ The Tauc plots are used to estimate the band gap energy (E_g_) of the samples, showing a consistent value of approximately 3.03 eV for all samples. This band gap value aligns closely with that of rutile TiO_2_, indicating that the primary optical properties are dominated by the TiO_2_ layer rather than Cu_2_O. The absence of a distinct Cu_2_O-related band gap (∼2.0–2.2 eV) is likely associated with the relatively low loading amount and finely dispersed surface distribution of Cu_2_O on the TiO_2_ NRs. Under these conditions, the dominant optical transition extracted from the Tauc analysis remains governed by the TiO_2_ phase. Therefore, the improved PEC performance observed for the Cu_2_O/TiO_2_ photoelectrodes is more likely attributed to enhanced interfacial charge separation and charge-transfer processes at the Cu_2_O/TiO_2_ heterojunction rather than significant band gap narrowing or strong visible-light absorption enhancement. In Figure 5B, the Cu_2_O/TiO_2_-5 min sample is partially overlapped by the Cu_2_O/TiO_2_-10 min curve due to their very similar optical absorption behavior and band gap characteristics.

Figure 5F presents the IPCE spectra of TiO_2_, Cu_2_O, and the Cu_2_O/TiO_2_-15 min photoelectrode measured over the wavelength range of 300–900 nm. The pristine TiO_2_ photoelectrode displayed a maximum IPCE value of approximately 62% at 340 nm, followed by a rapid decline beyond 400 nm, which is consistent with the wide bandgap nature of TiO_2_ that limits light absorption predominantly to the UV region.^36^^,^^46^ In contrast, the Cu_2_O photoelectrode exhibited photoresponse extending into the visible-light region up to approximately 600 nm owing to its narrower bandgap (∼2.0–2.2 eV).^36^^,^^62^ Remarkably, the Cu_2_O/TiO_2_-15 min heterojunction photoelectrode exhibited substantially enhanced photoresponse throughout the UV and visible regions. The maximum IPCE reached approximately 78% at 340 nm, which is significantly higher than that of pristine TiO_2_ and Cu_2_O. Furthermore, the heterostructure maintained appreciable photoactivity over a broad wavelength range extending to nearly 900 nm. The enhanced spectral response demonstrates the synergistic interaction between Cu_2_O and TiO_2_ and confirms the successful formation of an efficient heterojunction. The improved IPCE performance can be attributed to the favorable band alignment between p-type Cu_2_O and n-type TiO_2_. Similar enhancements in IPCE have been reported for Cu_2_O/TiO_2_ heterojunctions and other oxide-based photoelectrodes designed for solar hydrogen production^63^^,^^64^^,^^65^

Figures 6A and 6B illustrates the Nyquist plots of EIS for TiO_2_ and Cu_2_O/TiO_2_ photoelectrodes in two electrolytes: 1M KOH and 1M EtOH. The pristine TiO_2_ photoelectrode exhibits a relatively small semicircle diameter, indicating low interfacial charge-transfer resistance. In 1M KOH, the Cu_2_O/TiO_2_-15 min sample exhibits the smallest semicircle diameter among the Cu_2_O/TiO_2_ photoelectrodes, indicating relatively improved charge-transfer behavior compared to the Cu_2_O/TiO_2_-5 min and Cu_2_O/TiO_2_-10 min samples. This suggests that a 15-min deposition provides more favorable interfacial contact and heterojunction formation between Cu_2_O and TiO_2_. The Cu_2_O/TiO_2_-10 min sample shows a larger semicircle, reflecting higher resistance, while the Cu_2_O/TiO_2_-5 min sample displays the largest semicircle among the Cu_2_O/TiO_2_ photoelectrodes, indicating less efficient charge transfer. The comparison among the Cu_2_O/TiO_2_ samples suggests that increasing the Cu_2_O deposition time up to 15 min improves interfacial charge-transfer behavior and supports enhanced charge separation. Similarly, in 1M EtOH, the Cu_2_O/TiO_2_-15 min sample shows the smallest semicircle diameter among the Cu_2_O/TiO_2_ photoelectrodes, indicating relatively improved charge-transfer behavior. This behavior may be associated with improved interfacial contact and heterojunction formation after 15 min deposition. In contrast, the Cu_2_O/TiO_2_-10 min sample displays a larger semicircle, while the Cu_2_O/TiO_2_-5 min sample exhibits the largest semicircle, suggesting less efficient charge-transfer processes. Although the pristine TiO_2_ sample exhibits lower overall impedance, the enhanced PEC performance observed for the Cu_2_O/TiO_2_-15 min sample is likely related to improved interfacial charge separation and carrier utilization within the Cu_2_O/TiO_2_ heterostructure under illumination conditions. These results suggest that increasing the Cu_2_O deposition time up to 15 min improves the interfacial charge-transfer behavior and promotes more effective charge separation within the Cu_2_O/TiO_2_ heterostructure, supporting better photocurrent generation and overall PEC performance in the EtOH electrolyte. The equivalent circuit models for Cu_2_O/TiO_2_-15 min photoelectrodes in 1M KOH and 1M EtOH further support the observed PEC behavior. In 1M KOH, the circuit consists of two distinct charge transfer resistances (*R*_*p*_) of 177 Ω and 890 Ω, with corresponding constant phase elements (CPE) having *Y*_0_ values of 508 *μMho*·*s*^*N*^ and 2.47 *mMho*·*s*^*N*^. The higher *R*_*p*_ value for the second process suggests slower interfacial charge transfer, which is expected due to the hierarchical complexity of the system. Additionally, the solution resistance (*R*_*s*_) is relatively low at 5.56 Ω, indicating efficient ionic conductivity in the electrolyte. In 1M EtOH, the equivalent circuit shows *R*_*p*_ values of 653 Ω and 1.21 kΩ, which are higher compared to those in 1M KOH, reflecting greater charge transfer resistance in the ethanol system. The CPE parameters exhibit *Y*_0_ values of 79.4 *μMho*·*s*^*N*^ and 80.9 *μMho*·*s*^*N*^, with near-ideal capacitance behavior as indicated by *N* values of 0.99 and 0.916. The *R*_*s*_ in 1M EtOH is also higher at 18.8 Ω, likely due to the lower ionic conductivity of EtOH compared to aqueous KOH. Despite the higher resistances observed in 1M EtOH, the Cu_2_O/TiO_2_ photoelectrodes achieve higher PEC activity in this medium. This improved performance is attributed to ethanol acting as a hole scavenger, efficiently suppressing charge recombination and facilitating the generation of photocurrent.^58^^,^^60^ The presence of ethanol enhances the photoelectrode’s ability to separate and transport charges, resulting in higher PEC activity compared to the alkaline KOH system. This demonstrates that the electrolyte significantly influences the PEC performance, with ethanol providing an optimal environment for enhanced photocurrent generation. In Figure 6C, representing the M-S plots in 1M KOH, the Cu_2_O/TiO_2_-5 min shows the smallest slope, followed by the Cu_2_O/TiO_2_-10 min and Cu_2_O/TiO_2_-15 min. This indicates that the 5-min deposition time results in the highest carrier density among the samples in KOH, as a smaller slope is associated with a higher carrier density. Similarly, in Figure 6D, which shows the M-S plots in 1M EtOH, the Cu_2_O/TiO_2_-5 min again has the smallest slope, followed by the 10-min and 15-min samples, suggesting the same trend of decreasing carrier density with increasing deposition time in this electrolyte. Although the Cu_2_O/TiO_2_-5 min sample exhibits relatively higher carrier density based on the M-S analysis, this trend does not directly correspond to the EIS results or PEC performance. The discrepancy suggests that higher carrier density alone is insufficient to guarantee efficient interfacial charge transfer and photocurrent generation. In the present Cu_2_O/TiO_2_ system, shorter deposition times may produce thinner Cu_2_O layers with relatively higher apparent carrier density, but incomplete surface coverage and less effective heterojunction formation can limit charge separation and carrier transport across the interface. In contrast, the Cu_2_O/TiO_2_-15 min sample exhibits lower charge-transfer resistance, indicating improved interfacial charge separation and carrier utilization despite its relatively lower carrier density. These results suggest that the PEC performance is governed not only by carrier density, but also by heterojunction quality, interfacial charge-transfer behavior, and recombination dynamics.

**Figure 6:**
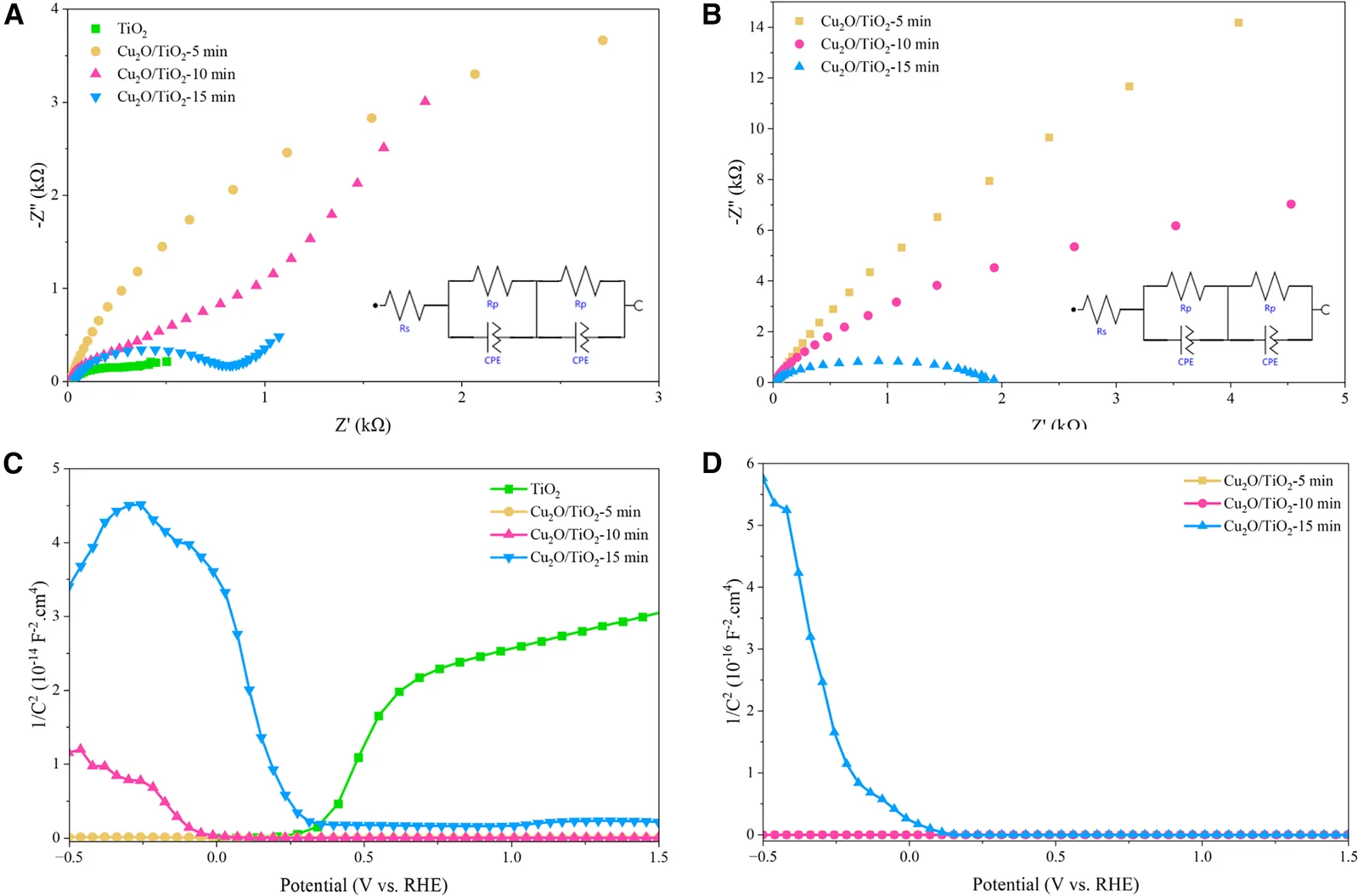
Nyquist plots of the electrochemical impedance spectra (EIS) data of Cu_2_O/TiO_2_ photoelectrodes in presence of (A) 1M KOH and (B) 1 M KOH and 1M EtOH electrolyte and Mott-Schottky (M-S) plots of Cu_2_O/TiO_2_ photoelectrodes in (C) 1M KOH and (D) 1M EtOH

### Photoelectrochemical performances of photoelectrodes

Figure 7 presents the LSV curves for Cu_2_O/TiO_2_ photoelectrodes with varying Cu_2_O deposition times (5, 10, and 15 min) under chopped light in different electrolytes (1M KOH and 1M EtOH). Figures 7A and 7B, the Cu_2_O/TiO_2_-15 min exhibits the highest photocurrent density among the samples, reaching 1.01 mA cm^−2^ and 1.30 mA cm^−2^ at 1.23 V vs. Ag/AgCl in 1M KOH and 1M KOH and 1M EtOH electrolytes, respectively. The photocurrent response under chopped illumination confirms the stable photoresponse behavior of the photoelectrodes. The prepared Cu_2_O/TiO_2_-15 min photoelectrode is comparable to other reported PEC systems of Cu_2_O/TiO_2_ photoelectrode^36^^,^^38^^,^^50^^,^^58^ (Table S1). The enhanced PEC performance of the Cu_2_O/TiO_2_-15 min sample is likely associated with improved interfacial charge separation and more favorable heterojunction formation between TiO_2_ and Cu_2_O, which promotes more efficient charge-transfer processes under illumination. The photocurrent response does not exhibit a strictly linear dependence on Cu_2_O deposition time, suggesting that the PEC activity is influenced not only by the amount of deposited Cu_2_O but also by the quality of heterojunction formation, interfacial charge-transfer behavior, and recombination dynamics. The relatively lower photocurrent densities observed for the Cu_2_O/TiO_2_-5 min and Cu_2_O/TiO_2_-10 min samples may be associated with incomplete heterojunction formation and less effective interfacial charge separation. For the Cu_2_O/TiO_2_-5 min sample, the shorter deposition time may result in insufficient Cu_2_O surface coverage on the TiO_2_ NRs, limiting the formation of effective charge-transfer pathways despite the relatively higher carrier density. In the case of the Cu_2_O/TiO_2_-10 min sample, although Cu_2_O deposition becomes more detectable, non-uniform surface distribution or localized interfacial recombination may adversely affect carrier transport and utilization. In contrast, the Cu_2_O/TiO_2_-15 min sample likely provides more favorable interfacial contact and heterojunction quality, resulting in more efficient charge separation and superior PEC performance. In 1M EtOH (Figure 7C), the Cu_2_O/TiO_2_ photoelectrodes exhibit enhanced PEC performance compared to 1M KOH, with the Cu_2_O/TiO_2_-15 min photoelectrode again achieving the highest photocurrent density. The Cu_2_O/TiO_2_-15 min photoelectrode exhibits approximately 1.7 times higher photocurrent than that of the pristine TiO_2_ photoelectrode. Importantly, the enhanced photocurrent response of the Cu_2_O/TiO_2_ photoelectrodes compared to pristine TiO_2_ was observed in both 1M KOH and 1M EtOH electrolytes, indicating that the Cu_2_O modification contributes to improved PEC activity even in the absence of ethanol assistance. The additional increase in photocurrent observed in EtOH is attributed to the sacrificial oxidation behavior of ethanol, which promotes hole consumption and suppresses charge recombination.^55^^,^^58^^,^^60^ These results suggest the potential of the Cu_2_O/TiO_2_-15 min photoelectrode for PEC water-splitting applications in ethanol-containing electrolytes.

**Figure 7:**
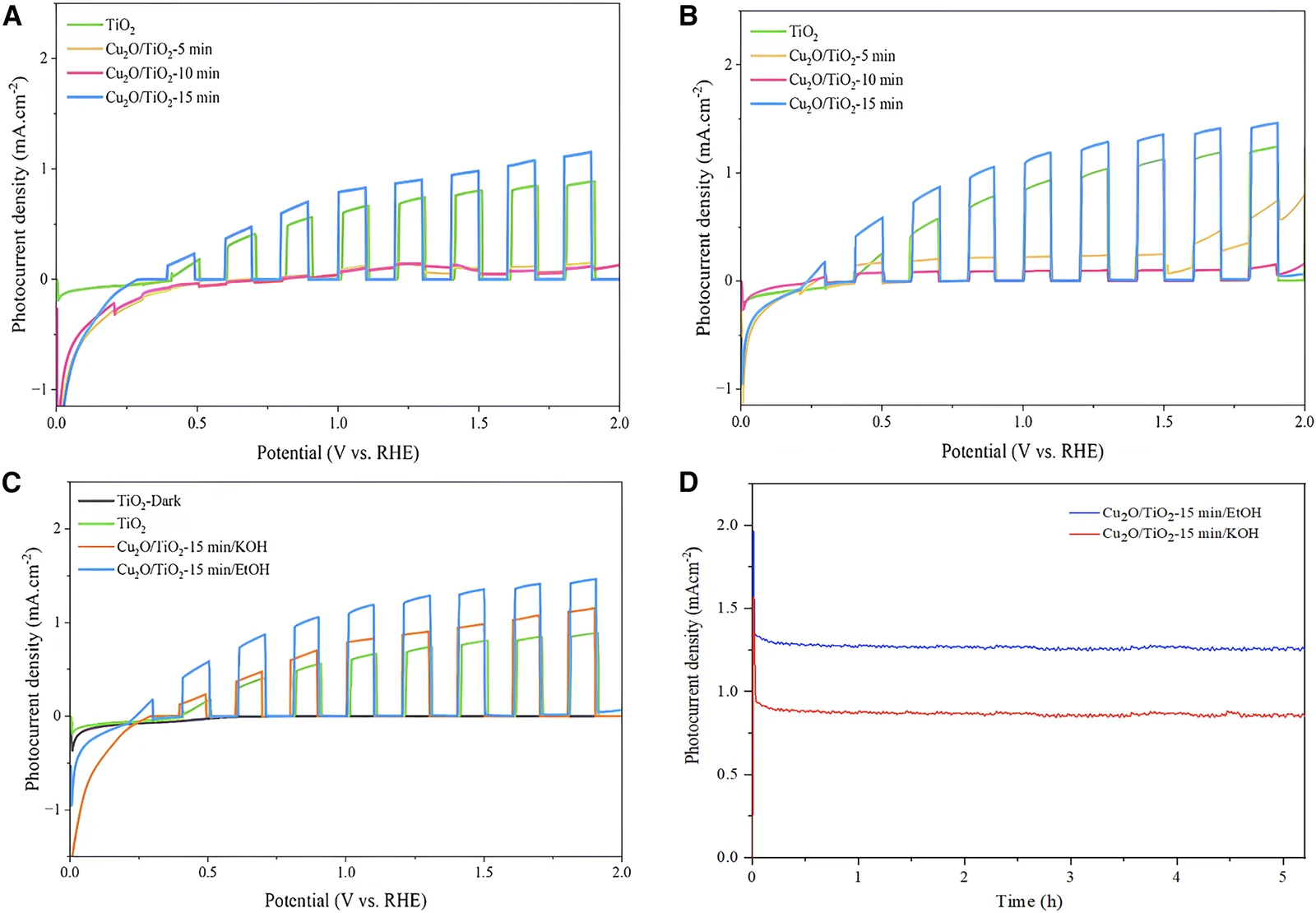
LSV curves for Cu_2_O/TiO_2_ photoelectrodes under chopped light in the presence of (A) 1M KOH and (B) 1M KOH and 1M EtOH electrolyte, (C) LSV curves for TiO_2_, Cu_2_O/TiO_2_-15 min in 1M KOH, and Cu_2_O/TiO_2_-15 min in 1M EtOH under chopped illumination and dark conditions, and (D) Long-term stability measurements of the Cu_2_O/TiO_2_-15 min photoelectrode under continuous illumination for 5 h in KOH and KOH containing ethanol

In addition to the enhanced photocurrent, the long-term stability of the Cu_2_O/TiO_2_ photoelectrode was evaluated by IT measurements under continuous illumination at 1.23 V vs. RHE for 5 h in 1 M KOH and 1 M KOH containing ethanol (EtOH) as shown in Figure 7D. Bare Cu_2_O photocathodes are known to undergo rapid photocorrosion, leading to severe photocurrent loss in alkaline solutions.^66^^,^^67^ In agreement with these reports, our bare Cu_2_O reference electrode in 1.0 M KOH also shows a pronounced photocurrent drop within the first hour of operation (Figure S1), consistent with our previous study.^55^^,^^68^ In contrast, the Cu_2_O/TiO_2_-15 photoelectrode exhibited excellent stability throughout the 5 h in both electrolytes. Notably, the Cu_2_O/TiO_2_ photoelectrode operated in the ethanol-containing electrolyte maintained a significantly higher photocurrent density throughout the entire testing period compared with that in pure KOH. After the initial transient decay, the photocurrent in both electrolytes remains nearly constant with negligible further loss, indicating good resistance to photocorrosion and stable interfacial charge-transfer kinetics.^37^^,^^38^ The improvement in stability is attributed to the Cu_2_O-TiO_2_ heterostructure, in which efficient charge separation and electron transfer from Cu_2_O to TiO_2_ suppress the self-reduction and oxidative degradation pathways that typically limit the operational lifetime of Cu_2_O-based photoelectrodes.^37^^,^^38^^,^^50^^,^^62^^,^^65^^,^^69^

The PEC hydrogen and oxygen evolution performances of the Cu_2_O/TiO_2_-15 min photoelectrode were evaluated under simulated solar irradiation in alkaline electrolyte without ethanol and in ethanol-containing electrolyte. The amounts of evolved H_2_ and O_2_ as a function of irradiation time are shown in Figures 8A and 8B. The theoretical gas production values calculated from the total transferred charge according to Faraday’s law (e^−^/2 for H_2_ and e^−^/4 for O_2_) are also included for comparison.

**Figure 8:**
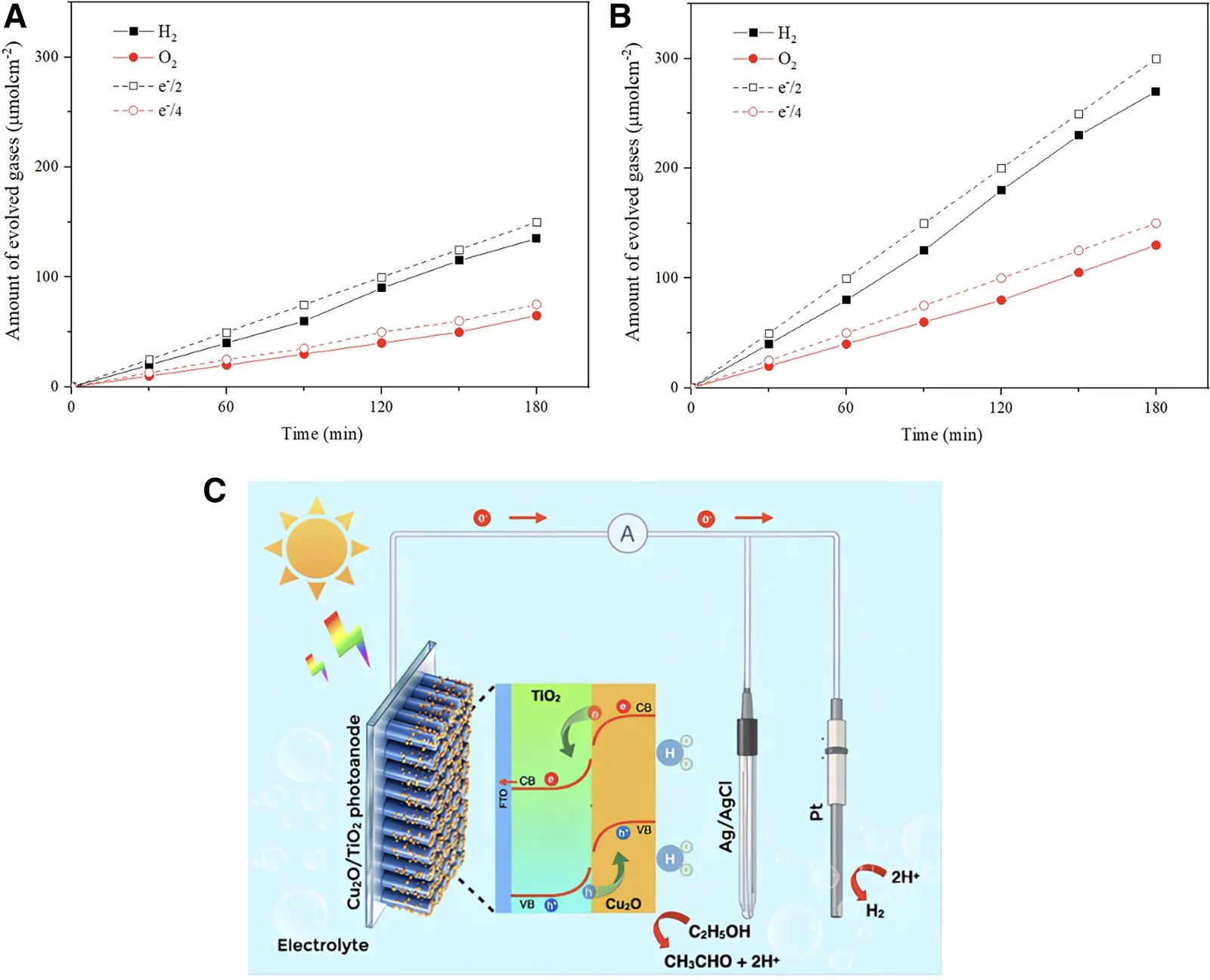
H_2_ and O_2_ evolution using the Cu_2_O/TiO_2_ photoelectrode under simulated solar irradiation in (A) KOH electrolyte and (B) KOH containing ethanol and (C) mechanism of Cu_2_O/TiO_2_ NRs photoelectrode in the PEC water splitting system

As shown in Figure 8A, the Cu_2_O/TiO_2_ photoelectrode operated in pure KOH generated H_2_ and O_2_ continuously throughout the 180 min reaction period. The amounts of H_2_ and O_2_ increased almost linearly with irradiation time, reaching approximately 135 and 65 μmol cm^−2^, respectively, after 180 min. The measured gas evolution rates closely followed the theoretical values calculated from the photocurrent, indicating efficient conversion of photogenerated charge carriers into chemical products. The H_2_/O_2_ ratio remained close to the stoichiometric value of 2:1 expected for overall water splitting, confirming that the generated gases originated predominantly from PEC water decomposition.

A significantly enhanced gas evolution performance was observed when ethanol was introduced into the electrolyte (Figure 8B). After 180 min of operation, the amounts of evolved H_2_ and O_2_ reached approximately 270 and 130 μmol cm^−2^, respectively, which are nearly two times higher than those obtained in the absence of ethanol. The enhancement in gas evolution is consistent with the chronoamperometric and photocurrent results discussed earlier, demonstrating that ethanol effectively promotes the PEC reaction. Ethanol acts as a sacrificial electron donor and is more readily oxidized than water due to its lower oxidation potential and more favorable reaction kinetics. As a result, the system operates as an ethanol-assisted alkaline PEC cell, where ethanol oxidation provides the majority of electrons for hydrogen evolution at the Cu_2_O/TiO_2_ photocathode, while still demonstrating that the electrode is capable of genuine water reduction in the absence of ethanol. Consequently, photogenerated holes are rapidly consumed by ethanol oxidation, suppressing surface charge recombination and increasing the availability of electrons for hydrogen evolution. Overall, the gas evolution measurements clearly demonstrate that the Cu_2_O/TiO_2_ photoelectrode exhibits efficient and stable solar-driven hydrogen production. The introduction of ethanol significantly enhances both H_2_ and O_2_ evolution rates, yielding approximately 2-fold higher gas production than that obtained in pure KOH.

## Discussion

Based on the above results, a proposed mechanism for PEC performance of a Cu_2_O/TiO_2_ photoelectrode within a three-electrode setup is illustrated in Figure 7D. In the PEC mechanism, an electrical potential is applied along with exposure to appropriately energized light onto the TiO_2_ NRs photoelectrode. When the interface between the electrolyte and the Cu_2_O/TiO_2_ is exposed to light with photon energies equal to or greater than the band gap (hν ≥ Eg), electron-hole pairs are generated. The photogenerated electrons in the CB of Cu_2_O migrate toward the TiO_2_ CB. From there, these electrons travel through the external circuit to the counter electrode (Pt), where they participate in H_2_ generation through reduction reactions. Concurrently, photogenerated holes in the VB of TiO_2_ migrate toward the Cu_2_O VB layer, where they facilitate oxidation reactions. In 1M EtOH and ethanol (C_2_H_5_OH) is oxidized to produce acetaldehyde (CH_3_CHO). The inherent electric field in the depletion region aids in the movement of some photogenerated charge carriers to the Cu_2_O/TiO_2_/electrolyte interface, where they facilitate the desired chemical reactions.^37^^,^^58^ These findings confirm that the synergistic combination of the Cu_2_O/TiO_2_ heterojunction and ethanol-assisted oxidation effectively improves charge separation, suppresses recombination, and promotes PEC hydrogen generation.

By synthesizing TiO_2_ NRs via a hydrothermal method and incorporating Cu_2_O to create a p-n heterostructure (Figure 9), the study aimed to improve interfacial charge separation and PEC performance. The results suggest that the enhanced activity primarily originates from more efficient charge carrier separation at the Cu_2_O/TiO_2_ interface, while the optical properties remain largely dominated by the TiO_2_ layer. After Cu_2_O electrodeposition onto TiO_2_, the Cu_2_O/TiO_2_-15 min sample exhibited the highest PEC performance in 1 M EtOH, achieving a significantly higher photocurrent density than pristine TiO_2_. This improvement is attributed to optimized interfacial charge transfer and reduced charge-transfer resistance. Furthermore, the comparison of performance between 1M EtOH and 1M KOH highlighted the potential of ethanol as a dual-function electrolyte, where its sacrificial agent properties contributed to higher photocurrent densities in the ethanol system. In summary, the Cu_2_O/TiO_2_ heterostructure photoelectrodes developed in this study represent a promising approach for PEC hydrogen production, particularly in EtOH electrolytes. The results demonstrated that the interaction between EtOH and the heterostructure offers unique advantages, including enhanced photocurrent density and effective charge separation. These findings pave the way for further exploration of renewable and sustainable sources for PEC water splitting, with ethanol serving as an effective medium for efficient hydrogen production.

**Figure 9:**
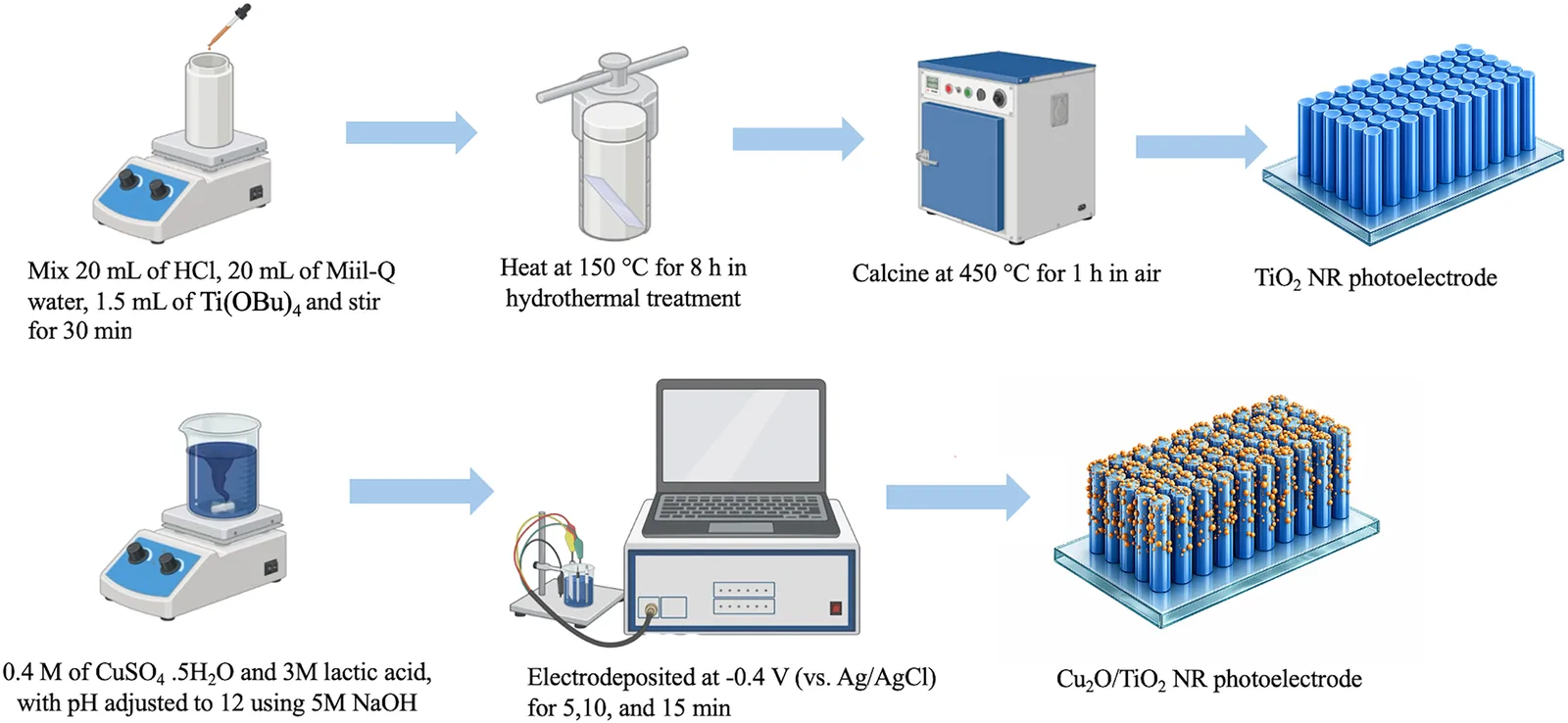
Schematic figure summarizing the fabrication processes for synthesizing Cu_2_O/TiO_2_ photoelectrodes

### Limitations of the study

Although the Cu_2_O/TiO_2_ heterostructure demonstrated enhanced PEC performance and good short-term stability, limitations remain in its preparation, characterization, and application. Regarding preparation, the Cu_2_O loading and surface coverage were mainly controlled by electrodeposition time, while the precise relationship among deposition conditions, Cu_2_O thickness, and interfacial structure has not been fully established. This limitation can be addressed by systematically optimizing the electrodeposition parameters and quantitatively controlling Cu_2_O loading and layer thickness to obtain a more uniform and reproducible heterostructure. Regarding characterization, the proposed interfacial charge-transfer mechanism is primarily supported by structural, optical, and electrochemical analyses, whereas direct evidence of charge-carrier dynamics at the Cu_2_O/TiO_2_ interface is still limited. Regarding application, the long-term durability of the photoelectrode has not yet been established, and the enhanced hydrogen evolution observed in the ethanol-containing electrolyte is partly associated with sacrificial ethanol oxidation. Longer-term stability testing under continuous and repeated illumination, together with post-reaction structural and surface characterization, is therefore required to assess photocorrosion and material degradation. In addition, further optimization under sacrificial-agent-free conditions and evaluation under realistic solar irradiation and scalable electrode configurations will be necessary to determine the practical potential of Cu_2_O/TiO_2_ photoelectrodes for sustainable solar-driven hydrogen production.

## Resource availability

### Lead contact

Requests for further information and resources should be directed to and will be fulfilled by the lead contact, Piangjai Peerakiatkhajohn (piangjai.pee@mahidol.ac.th).

### Materials availability

This work did not generate new unique reagents. All materials and methods used for data generation and analysis have been mentioned in the article.

### Data and code availability

All data reported in this article will be shared by the lead contact upon request. This article does not report original code. Any additional information required to analyze the data reported in this article is available from the lead contact upon request.

## Acknowledgments

This research project is supported by Mahidol University (MU’s Strategic Research Fund: 2023). The authors would like to acknowledge the Faculty of Environment and Resource Studies, and Mahidol University-Frontier Research Facility (MU-FRF), Mahidol University and National Nanotechnology Center (NANOTEC), Thailand for providing the experimental facilities and technical support.

## Author contributions

Conceptualization, S.K., S.T., and P.P.; methodology, S.K. and P.P.; validation, S.K., T.B., S.T., J.-H.Y., M.L., and P.P.; formal analysis, S.K., T.B., S.T., J.-H.Y., M.L., and P.P.; investigation, S.K., T.B., S.T., J.-H.Y., M.L., and P.P.; resources, T.B., S.T., M.L., and P.P.; visualization, S.K. and P.P.; writing – original draft preparation, S.K., and P.P.; writing – review and editing, S.K., T.B., S.T., J.-H.Y., M.L., and P.P.; and supervision, P.P.

## Declaration of interests

The authors declare no competing interests.

## Declaration of generative AI and AI-assisted technologies in the manuscript preparation process

During the preparation of this work, the authors used ChatGPT (OpenAI) in order to improve the clarity, grammar, readability, and academic style of the manuscript. After using this tool or service, the authors reviewed and edited the content as needed and take full responsibility for the content of the publication.

## STAR★Methods

### Key resources table

**Table:** 

REAGENT or RESOURCE	SOURCE	IDENTIFIER
**Chemicals**		

Titanium (IV) butoxide (Ti(OBu)_4_), reagent grade, 97%	Sigma-Aldrich	CAS 5593–70–4
Hydrochloric acid (HCl), ACS reagent, 37%	Sigma-Aldrich	CAS 7647–01–0
Copper (II) sulfate pentahydrate (CuSO_4_·5H_2_O), ACS reagent, ≥98%	Sigma-Aldrich	CAS 7758–99–8
Lactic acid solution, ACS reagent, ≥85% in H_2_O	Sigma-Aldrich	CAS 50–21–5
Sodium hydroxide (NaOH), ACS reagent, ≥97%, pellets	Sigma-Aldrich	CAS 1310–73–2
Potassium hydroxide (KOH), ACS reagent, ≥85%, pellets	Sigma-Aldrich	CAS 1310–58–3
Ethanol, ACS reagent, ≥99.5%	Sigma-Aldrich	CAS 64–17–5
Acetone, ACS reagent, ≥99.5%	Sigma-Aldrich	CAS 67–64–1

**Software and algorithms**		

Excel	Microsoft Office	https://www.office.com
Origin Pro 2026	OriginLab	https://www.originlab.com

**Other**		

Fluorine-doped tin oxide (FTO) coated glass (15 × 40 × 2.3 mm, 15 Ω sq^−1^)	Dyesol Glass	Cat MS200222
Field-emission scanning electron microscope (FE-SEM)	JEOL	JSM-IT500LA
Transmission electron microscope (TEM/HRTEM)	JEOL	JEM-2100 Plus
X-ray diffractometer	Bruker	D2 PHASER
Raman microscope	HORIBA	XploRA PLUS
FTIR spectrometer	Thermo Fisher Scientific	Nicolet iS50
UV-visible spectrophotometer	Shimadzu	UV-2600
Potentiostat	Metrohm Autolab	M204
Solar simulator, AM 1.5G	Newport	LCS-100

### Experimental model and study participant details

This study does not involve biological models.

### Method details

#### Synthesis of TiO_2_ NR photoelectrode

TiO_2_ nanorod (TiO_2_ NR) photoelectrodes were grown on FTO-coated glass substrates via a hydrothermal method. Prior to synthesis, the FTO substrates were ultrasonically cleaned in ethanol, acetone, and Milli-Q water for 15 min each, then dried under a nitrogen (N_2_) stream. The precursor solution was prepared by mixing 20 mL of hydrochloric acid (HCl) with 20 mL of Milli-Q water, followed by the dropwise addition of 1.5 mL titanium butoxide (Ti(OBu)_4_). After stirring for 30 min to ensure a homogeneous 3.75% (v/v) concentration, the solution and the FTO substrate (conductive side facing downward) were transferred to a 50 mL Teflon-lined stainless-steel autoclave. Hydrothermal growth was conducted at 150°C for 4, 6, or 8 h. The resulting films were rinsed with Milli-Q water, dried, and subsequently calcined at 450°C for 1 h in air to improve crystallinity.

#### Synthesis of Cu_2_O/TiO_2_ NR photoelectrode

Cu_2_O was decorated onto the TiO_2_ NR arrays via a potentiostatic electrodeposition technique. The electrolyte was prepared by dissolving 0.4 M CuSO_4_⋅5H_2_O in 3 M lactic acid, with the pH subsequently adjusted to 12 using a 5 M NaOH solution. Continuous stirring resulted in a stabilized dark blue precursor complex. A standard three-electrode configuration was employed for the deposition, utilizing an electrochemical workstation at a constant potential of −0.4 V (vs. Ag/AgCl). To systematically vary the Cu_2_O loading, the deposition time was set at 5, 10, and 15 min. Finally, the resulting Cu_2_O/TiO_2_ NR photoelectrodes were rinsed with Milli-Q water and dried in air as shown in Figure 9.

#### Characterization

The physicochemical properties of the synthesized samples were systematically investigated using a suite of advanced characterization techniques. Surface morphology and particle size were examined via field-emission scanning electron microscopy (FE-SEM, JEOL JSM-IT500LA) at an accelerating voltage of 10 kV. Elemental composition and mapping were performed using energy-dispersive X-ray spectroscopy (EDS) integrated with the FE-SEM system at 15 kV. For more detailed structural analysis, transmission electron microscopy (TEM) and high-resolution TEM (HRTEM) were conducted on a JEOL 2100 Plus (200 keV) equipped with EDX capabilities. Crystal structures were determined by X-ray diffraction (XRD, BRUKER D2 PHASER) using Cu−Kα radiation (λ = 1.5406 Å) in the 2θ range of 20°–80°. The crystalline phases of the TiO_2_ and Cu_2_O/TiO_2_ samples were further validated by Raman spectroscopy (HORIBA XPloRA PLUS) using a 523 nm argon-ion laser (200–1000 cm^−1^). Functional groups were identified using Fourier transform infrared spectroscopy (FTIR, Thermo Fisher Scientific iS50) from 4000 to 650 cm^−1^, while optical properties and bandgap energies were evaluated via UV-Visible spectrophotometry (Shimadzu UV-2600).

#### Photoelectrochemical measurement

PEC evaluations of the TiO_2_-based photoanodes were conducted using a Metrohm Autolab M204 potentiostat/galvanostat coupled with a BOOSTER10A. A standard three-electrode configuration was employed, with the TiO_2_ sample as the working electrode, a platinum (Pt) wire as the counter electrode, and Ag/AgCl (3 M KCl) as the reference electrode. The measurements were performed in a 1 M KOH electrolyte under simulated AM 1.5G solar illumination (100 mW/cm^2^, Newport LCS-100). Potentials were converted to the reversible hydrogen electrode (RHE) scale according to the Nernst equation: VRHE = VAg/AgCl +0.059 × pH + V^°^_Ag/AgCl_ (V^°^_Ag/AgCl_ = 0.1976 V). Linear sweep voltammetry (LSV) was recorded from 0 to 2.5 V vs. RHE at a scan rate of 0.01 V/s under both continuous and chopped illumination. Transient photocurrent responses (I−t) were monitored over 30 s on/off cycles. Electrochemical impedance spectroscopy (EIS) was carried out in the dark using a 5 mV amplitude over a frequency range of 0.1 Hz–10 kHz. Furthermore, cyclic voltammetry (CV) was performed between 0.4 and 2 V vs. RHE (0.1 V/s), and Mott-Schottky (M-S) plots were derived in the dark at a fixed frequency of 1 kHz to evaluate the flat-band potential and carrier density.

### Quantification and statistical analysis

Photoelectrochemical measurements were independently performed three times under identical experimental conditions to ensure the reproducibility of the results. The reported photocurrent density values represent the mean of three independent measurements. The photoelectrochemical curves presented in the figures are representative measurements; therefore, error bars are not displayed on the curves.

The optical bandgap energies were determined from UV-Vis absorption data using Tauc plots by extrapolating the linear region of the plots to the photon-energy axis. Carrier densities were calculated from the slopes of the linear regions of the Mott-Schottky plots according to the Mott-Schottky relationship.

SEM images were used to evaluate the surface morphology and structural characteristics of the prepared electrodes. No statistical particle-size distribution analysis was performed.

No statistical significance tests or analysis of variance (ANOVA) were performed in this study. The quantitative analyses were primarily used to characterize and compare the optical, electronic, and photoelectrochemical properties of the prepared photoelectrodes.
