# Supplementary material for: Stable Cu2O/TiO2 nanorod photoelectrodes for enhanced photoelectrochemical water splitting in ethanol-assisted systems
Source: iScience. 2026 Oct 28;29(11):117997. doi: 10.1016/j.isci.2026.117997 (PMC13738174; doi:10.1016/j.isci.2026.117997)
Supplement: Supplementary file 1 [file mmc1.pdf]

iScience, Volume *29*

Supplemental information

Stable Cu_2O/TiO_2 nanorod photoelectrodes for enhanced photoelectrochemical water splitting in ethanol-assisted systems

Srijitra Khanpakdee, Teera Butburee, Supphasin Thaweesak, Jung-Ho Yun, Miaoqiang Lyu, and Piangjai Peerakiatkhajohn

Supplemental information

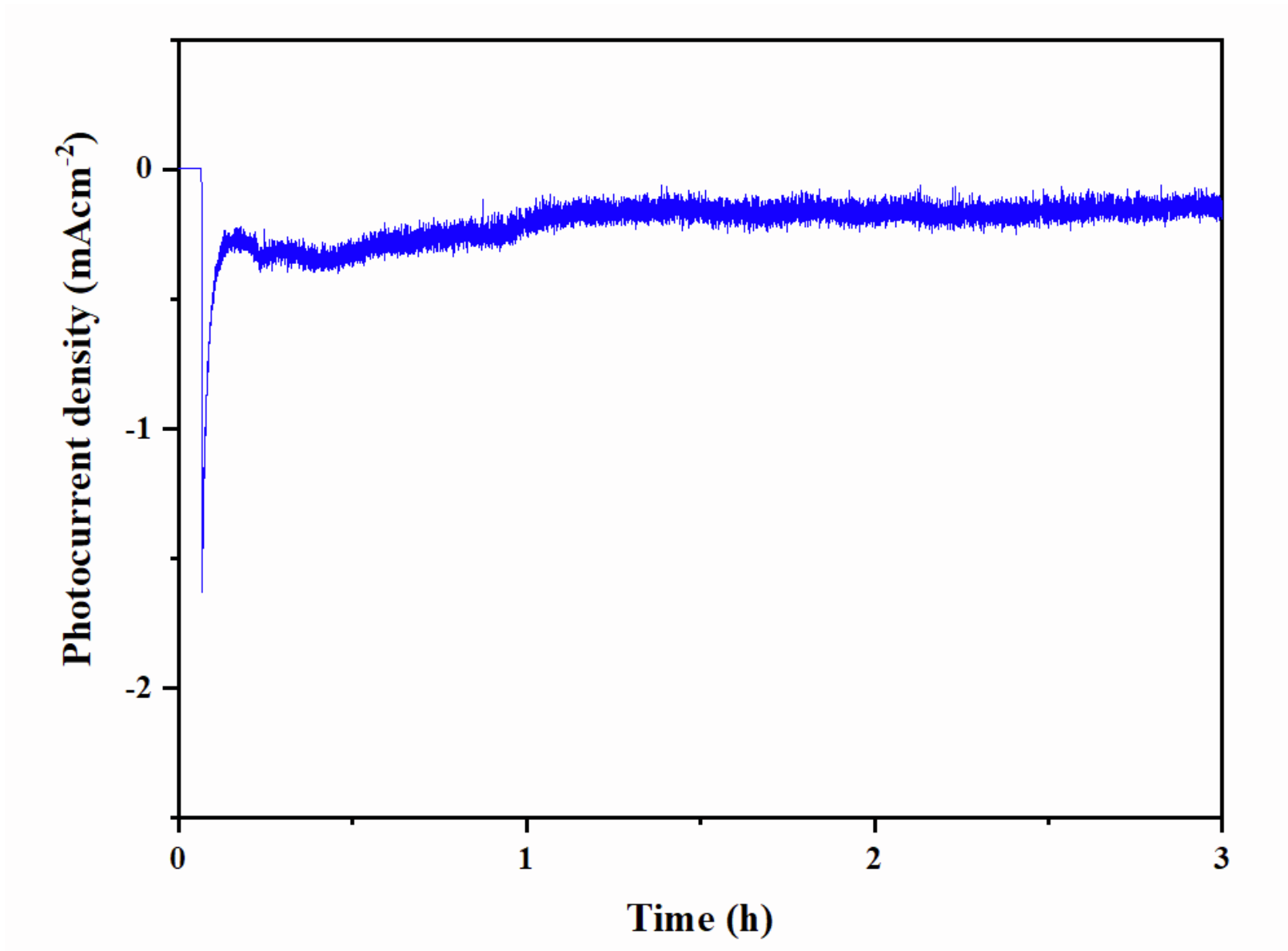

Figure S1 Chronoamperometric (I–t) curves of the bare FTO/Cu_2O photoelectrode under AM 1.5G illumination in 1.0 M KOH, showing a rapid decay of photocurrent within the first hour of operation and confirming the poor stability of pristine Cu_2O photocathodes in alkaline electrolyte.

Table S1 Comparison of fabrication methods and photoelectrochemical performance of Cu_2O/TiO_2 heterostructure photoelectrodes reported in recent studies.

Photoelectrode	Method	Electrolyte	Light Source /Intensity	Photocurrent Density (mA.cm-²)	Ref.
Cu_2O/TiO_2 Nanorods	Hydrothermal/ Electrodeposition	1 M KOH + 1M Ethanol	AM 1.5G (100 mW.cm^{-2})	1.30 mW.cm^{-2} 1.23 V vs. RHE	This work
Cu_2O/TiO_2 Nanorods	Hydrothermal/ Electrodeposition	1 M KOH	AM 1.5G (100 mW.cm-^{2})	1.01 mW.cm^{-2} 1.23 V vs. RHE	This work
Cu_2O/TiO_2 Nanosheets	Hydrothermal / Electrodeposition	0.5 M Na_2SO_4	AM 1.5G (100 mW.cm^{-2})	0.14 mW.cm^{-2} 0.2 V vs. Ag/AgCl	Wang et al.,2025
K-doped n- Cu_2O	Electrodeposition	0.5 M Na_2SO_3	200 W Tungsten/ Halogen lamp	0.047 mW.cm^{-2} 0.2 V vs. Ag/AgCl	Abdelfatah et al.,2023
Cu_2O/STNA (Short TiO_2 Nanotube Array)	Sonoelectro- chemical deposition method (SED)	0.1 M Na_2SO_4	350 W Xe lamp	0.5 mW.cm^{-2} 0.2 V vs. Ag/AgCl	Liu et al.,2023
Cu_2O/TiO_2 (R/T) (Nanoring/ Nanotube)	Two-step anodic oxidation / Thermal decomposition	Not explicitly specified	Not explicitly specified	0.023 mW.cm^{-2}	Yu et al.,2023
